# Analysis of Forensic Casework Utilizing Infrared Spectroscopic Imaging †

**DOI:** 10.3390/s16030278

**Published:** 2016-02-24

**Authors:** Adam Lanzarotta

**Affiliations:** FDA Forensic Chemistry Center, Cincinnati, OH 45237, USA; adam.lanzarotta@fda.hhs.gov; Tel.: +1-513-679-2700

**Keywords:** infrared spectroscopic imaging, forensic science, adulteration, dietary supplement, melamine cyanurate

## Abstract

A search of the current scientific literature yields a limited number of studies that describe the use of Fourier transform infrared (FT-IR) spectroscopic imaging for the analysis of forensic casework, which is likely due to the fact that these instruments are fairly new commodities to the field of analytical chemistry and are therefore not yet commonplace in forensic laboratories. This report describes recent forensic case studies that have used the technique for determining the composition of a wide variety of multi-component sample types, including animal tissue sections for toxic inclusions, drugs/dietary supplements, an antibiotic with an active pharmaceutical ingredient (API) present as several different salt forms, an adulterated bulk API, unknown trace powders for illicit drugs and an ophthalmic solution suspected of being adulterated with bleach.

## 1. Introduction

The ability to examine samples in their native state is one of the most significant advantages offered by infrared spectroscopy compared to most other analytical techniques. Examinations using this approach are generally fast, require minimal sample preparation and often do not require the use of solvents. Although it is not nearly as sensitive as chromatographic/mass spectrometric approaches, infrared spectroscopy can be conducted on a wide variety of sample types (e.g., finished dosages, as well as packaging), is useful for identifying both active ingredients and excipients and is able to differentiate polymorphs, as well as salt forms from free acids and bases. The technique can also be advantageous for analytes with higher and lower m/z ratios than instrument cutoffs, analytes with non-descript mass spectra and analytes not readily soluble in solvents used for general screens.

Regarding macro-infrared spectroscopy, which typically uses a sampling aperture on the order of mm and a single element detector, many of these advantages are often limited to pure compounds or analytes present above 1% in a non-interfering matrix [1]. On the other hand, these advantages are not always realized for determining the composition of a multi-component sample because the individual particle sizes are relatively small compared to the sampling aperture. The result is typically a complex mixture spectrum that requires spectral subtraction to identify individual components. Although spectral subtractions can be useful for identifying multiple ingredients, this approach is only effective until subtraction residuals dominate the spectrum, at which point a direct comparison to a standard becomes difficult. Multi-component samples can be manually separated, and individual particles can be identified using infrared microspectroscopy, which also utilizes a single-element detector, but with a smaller aperture that is closer to the size of individual particles (down to about 10 μm). Unfortunately, infrared microspectroscopic analyses are often time consuming, require a skilled analyst and can be difficult when the sample contains small particles or several particle types with a similar morphology.

In many cases, a more efficient and effective approach includes infrared spectroscopic imaging, which uses a multi-channel detector to collect an infrared spectrum at each spatial location in a two-dimensional region of interest. The size of each spatial element at the sample plane is defined by the size of the pixels on the detector and optics of the instrument. In many cases, the instrument can be configured so that the pixels are smaller than the particles of interest, in which case the spatial resolution of the measurement becomes either diffraction limited or, more likely, particle size limited. As a result, particles that are physically separated from one another often yield spectra that are characteristic of nearly pure compounds, thereby providing the ability to identify individual ingredients of a multi-component sample.

In a sense, the advent of infrared spectroscopic imaging turned the most significant disadvantage of macro-infrared spectroscopy, the ability to identify individual ingredients in a multi-component sample, into an advantage [2]. In addition to the benefits enjoyed by all infrared techniques, with infrared spectroscopic imaging, many analytes can be detected in the presence of each other, and low and high concentration analytes can often be detected in a single measurement, all without having to modify solvents, concentrations, columns, *etc.*, such as with chromatographic and mass spectrometric techniques. In fact, a single set of parameters is often suitable for most samples. Although detection limits for this approach are still several orders of magnitude higher than chromatographic and mass spectrometric techniques, its versatility makes it ideal for use as a screening method early in an investigation.

Infrared spectroscopic imaging has recently been employed for a few forensic analyses requiring the ability to identify individual ingredients spatially isolated from one another within a multi-component sample [3,4,5,6,7,8,9,10,11,12,13,14,15,16,17,18,19,20,21]. For example, researchers have employed this approach to detect trace evidence, such as drugs and explosives between the ridges of latent fingerprints [3,5,7,11,12,13], as well as for the analysis of cross-sectioned paint chips [4], bi-component fibers [6], counterfeit tablets [8], intersecting lines in questioned documents [10], counterfeit bank notes [18] and gunshot residue [19]. Forensic analyses using infrared spectroscopic imaging at the Forensic Chemistry Center (FCC) have included determining the composition of combination drugs (those with more than one API) [1], suspected counterfeit tablets [15], illicit pharmaceuticals [16], dietary supplements [17], human autopsy tissue extractions [20] and cross-sectioned suspected counterfeit packaging materials, such as adhesive labels, foil safety seals and cigarette tear tape [21]. It was the intent of this manuscript to describe additional, yet to be published analyses conducted by FCC and to highlight some additional advantages of this instrumental approach that has the ability to answer forensically-relevant questions that cannot be readily answered, or answered as easily, using most other techniques.

## 2. Experimental Results

### 2.1. Background

FCC is responsible for the analysis of compromised FDA-regulated products resulting from adulteration, tampering, counterfeiting and diversion. Over the last several years, FCC analysts have employed infrared spectroscopic imaging to examine a wide variety of multi-component samples for forensic casework due to the advantages that it offers compared to more established techniques. In addition to the studies in the open literature described above [1,15,16,17,20,21], FCC analysts have found that the technique is valuable for the analysis of a wide variety of sample types, including tissue cross-sections for toxic inclusions, several different multi-API drug formulations/dietary supplements, samples with an API present as several different salt forms, bulk ingredients for impurities, unknown trace powders with multiple ingredients and tampered drug solutions.

### 2.2. Analysis of Animal Tissue Cross-Sections for Toxic Inclusions

Economically-motivated adulteration has plagued the world’s food supply for centuries [22]. Recently, wheat gluten and rice protein bulk pet food ingredients were substituted with less expensive alternatives, such as wheat flour [23,24,25,26]. In order to pass screening tests that measured total nitrogen content as a surrogate for protein, violators added melamine and other nitrogen-rich triazine compounds to the wheat flour. Hydrogen bonding between melamine and cyanuric acid formed melamine cyanurate, a toxic compound that led to renal failure and death of hundreds of dogs and cats that consumed the pet food. Melamine cyanurate crystal inclusions have been detected in animal kidney tissue using Raman mapping [27], which has helped veterinarians determine the cause of death in pets that consumed the tainted food. Although Raman mapping is non-destructive, the technique is typically limited to small sample areas to achieve a sufficient tradeoff between resolution, sensitivity and acquisition time. Larger, more representative images can be collected in shorter timeframes using FT-IR spectroscopic imaging, which was first demonstrated for detecting melamine cyanurate in cat tissue sections by Marcott *et al.* [28].

Attenuated total reflection (ATR) is the most commonly-employed imaging mode at the FCC because it allows for solid state analysis, requires little-to-no sample preparation, provides an improvement in spatial resolution compared to measurements performed in air by a factor equal to the refractive index of the internal reflection element (IRE), is typically sample thickness independent and typically yields photometrically-accurate spectra. However, this approach is destructive due to the fact that it requires intimate contact with the sample, and its image area is limited by the size of the IRE (only ~400 μm × 400 μm for the instrument employed by this study). On the other hand, regarding the interaction of light with the sample, transmission and reflection/absorption (R/A) infrared methods are minimally destructive, and their image areas are limited only by the boundaries of the stage motor (>10 mm × 10 mm for the instrument employed by this study). These approaches, however, are prone to spectral distortions that yield non-photometrically accurate spectra, which make direct comparison to a standard difficult.

**Figure 1:**
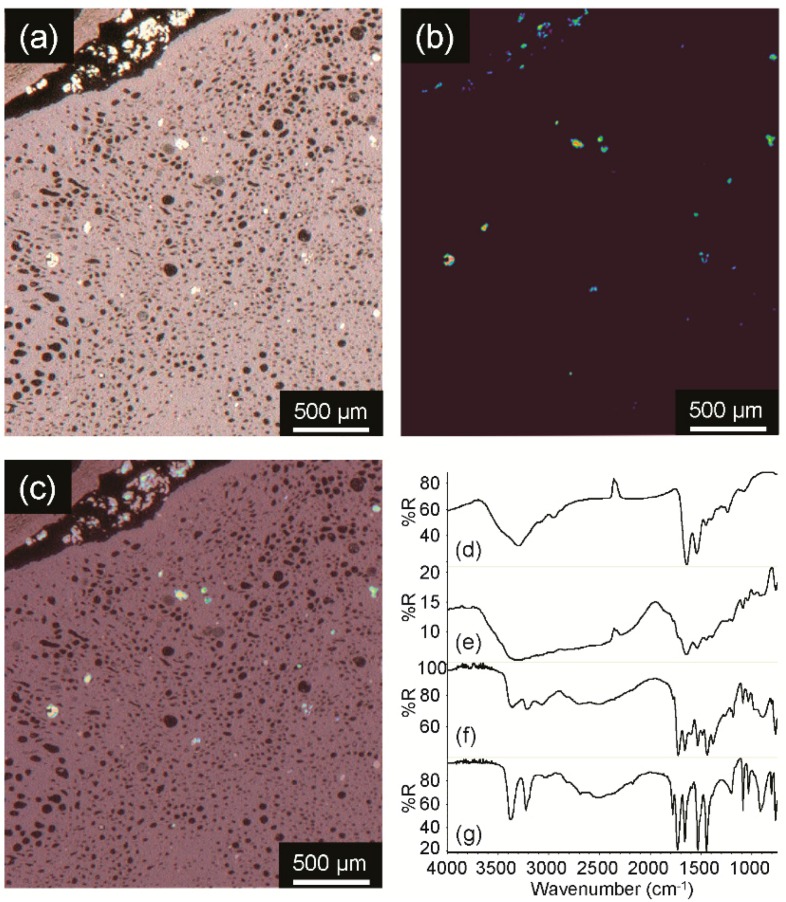
Visible image (**a**); R/A correlation image for melamine cyanurate (**b**); overlay image of (a) and (b), (**c**); representative R/A spectrum from a low correlation region (**d**); representative R/A spectrum from a high correlation region (**e**); ATR spectrum from a high correlation region (**f**); and ATR spectrum of a melamine cyanurate pure compound reference standard (**g**).

It was therefore of interest to employ a method that simultaneously retained the spectral fidelity of ATR and the minimally-destructive capabilities of transmission and R/A approaches. The approach used by the FCC consists of screening a large region in transmission or R/A mode followed by collecting ATR spectra on a limited number of suspect particles with a “drop down” ATR accessory with a diameter of only 100 μm, which is similar to the approach described by Gupper *et al.* [29]. In this case, a large area can be imaged, and the ATR accessory only makes contact with a few particles so that the remaining sample can be left unaltered. For example, Figure 1a is a photomicrograph of a cross-sectioned kidney tissue section from a cat that consumed adulterated pet food. The sample was received on a low-E (tin oxide-coated, infrared reflecting) microscope slide, which prohibited examination by transmission, but was ideal for R/A. An infrared image was therefore collected in R/A mode on the entire viewing area. A correlation search for melamine cyanurate yielded the image in Figure 1b, where bright regions correspond to pixels with spectra that have a high correlation to the target compound. Figure 1a,b has been overlaid in Figure 1c, which indicates that the high correlation regions correspond to bright circular regions in the tissue. The representative R/A infrared spectrum from a low correlation region provided in Figure 1d exhibited amide A, I and II absorptions characteristic of protein at approximately 3300 cm^−1^, 1640 cm^−1^ and 1542 cm^−1^, respectively. The representative R/A infrared spectrum from a high correlation region provided in Figure 1e is clearly not consistent with that of the tissue, although its poor quality prohibited further identification. First, the spectrum resides between 20% and 5% R, which is well outside an appropriate range. Second, the spectrum exhibits a sloping baseline, and third, significant band broadening is observed to the point where resolution between two adjacent bands is not achieved. As a result, the stage was positioned on a high melamine-cyanurate correlation region, and the “drop-down” ATR accessory was lowered until it made contact with the sample. The resulting ATR spectrum, Figure 1f, exhibited a non-sloping baseline, a sufficient dynamic range and narrower peaks that were consistent with those of the melamine cyanurate standard reference spectrum provided in Figure 1g. The peak broadening in the case of Figure 1f was likely due to an altered crystalline structure from interactions with the cat’s digestive tract.

### 2.3. Analysis of Multi-API Drug Formulations/Dietary Supplements

A large volume of samples examined by the FCC involve suspected counterfeit, diverted or misbranded pharmaceuticals and/or dietary supplements. In each case, the requesting case agent wants to know, in addition to whether or not the product is counterfeit, if the finished dosage contains labeled and/or unlabeled API(s). APIs in single-API formulations can typically be identified easily by macro-ATR-FT-IR spectroscopy followed by spectral subtractions or an extraction [14]. However, spectral subtractions and extractions are not typically effective for identifying each API in a multi-API formulation (e.g., combination drugs, dietary supplements) due to spectral distortions resulting from the subtraction process and co-solubility, respectively. In the past, APIs in multi-API formulations were therefore typically identified using a technique with an upfront separation stage, such as gas chromatography with mass spectrometric detection (GC/MS) or liquid chromatography with mass spectrometric detection (LC/MS). However, now that FT-IR spectroscopic imaging is available, analytes spatially isolated from one another can be identified in a solid state with as little as 25 ng of sample [15]. Although it has already been determined that this technique is effective for identifying APIs in multi-API formulations, such as combination drugs [1], the versatility of the technique has not yet been reported. Specifically, a wide variety of products can be examined with a single set of collection parameters. For example, APIs in the combination drugs and dietary supplements listed in Table 1 were all detected under the same conditions using ATR-FT-IR spectroscopic imaging: a 1.5-mm germanium hemisphere IRE, 400 μm × 400 μm image area, 1.56 μm × 1.56 μm pixel resolution, 16 cm^−1^ spectral resolution, one scan per pixel and 13-min collection time. In fact, all ATR imaging analyses conducted in this manuscript and several already in the open literature were also collected with these same parameters [1,15,16,17]. It should be noted that when using ATR, the image area is limited by the size of IRE, so the detection limit of this approach is dictated by the particle size of the matrix (assuming the particles are larger than the diffraction-limited focused beam diameter, which is the case for most samples). Therefore, in order for this approach to be effective, the sample must contain particles that are significantly smaller than the image area. The relationship between particle size and detection limit of the ATR-FT-IR imaging instrument used in this study has been described in more detail elsewhere.[1] As a point of reference, the instrument yielded a practical detection limit of 220 ppm for a sample with an average matrix particle size of 23.4 μm.

### 2.4. Analysis of Samples with an API Present as Several Different Salt Forms

**Figure 2:**
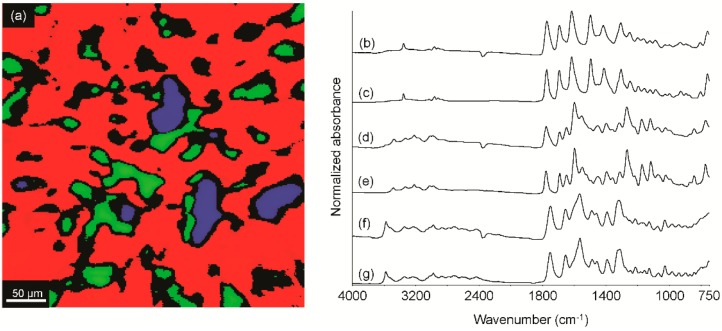
ATR-FT-IR image of the suspect product (**a**); and representative spectra from the blue (**b**); green (**d**) and red (**f**) regions compared to pure compound reference spectra of penicillin G sodium salt (**c**); penicillin G procaine salt hydrate (**e**) and penicillin G benzathine salt (**g**).

Using LC/MS, an unknown powder was found to contain penicillin G, benzathine and procaine. However, using this approach, it was not possible to determine if these three ingredients were present independently or if the sample contained benzathine and/or procaine salts of penicillin G (or a combination thereof). Due to the advantages of infrared spectroscopic imaging described above, the sample was therefore further examined using this approach (Figure 2a).

Representative spectra from the blue (Figure 2b), green (Figure 2d) and red (Figure 2f) regions were peak-for-peak matches with penicillin G sodium salt (Figure 2c), penicillin G procaine salt hydrate (Figure 2e) and penicillin G benzathine salt (Figure 2g), respectively. Black regions in this image and those of the remaining images in this manuscript correspond to mixture spectra or noise spectra (either from poor contact or absence of the analyte). These data demonstrate the efficacy of FT-IR spectroscopic imaging for determining salt forms of multi-API formulations, which was important, because each has a different impact on the body. These results demonstrate the ability of FT-IR spectroscopic imaging to overcome potential pitfalls characteristic of other, more commonly-employed analytical instruments.

### 2.5. Analysis of Bulk Ingredients for Impurities

**Figure 3:**
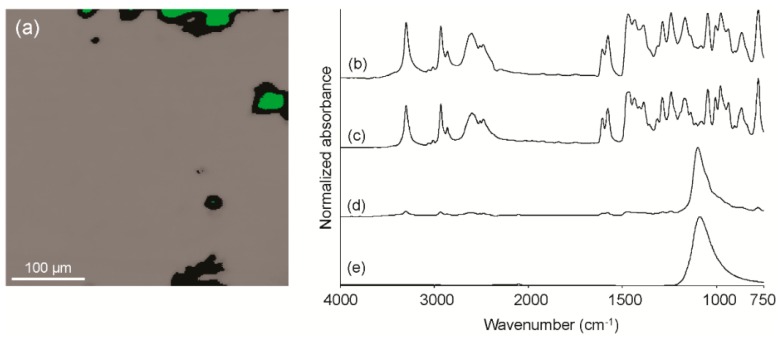
ATR-FT-IR image of the bulk API (**a**); representative spectrum from the gray region (**b**); pure compound reference spectrum of the API (**c**); representative spectrum from the green region (**d**); pure compound reference spectrum of sodium sulfate (**e**).

Using high performance liquid chromatography with ultraviolet detection (HPLC-UV), a bulk API recently received by this laboratory was found to be only 70.4% pure and therefore did not conform to United States Pharmacopoeia (USP) assay requirements. However, other than the API itself, no other compounds were detected in the chromatogram using this approach. The sample was therefore further examined using FT-IR spectroscopic imaging for the presence of compounds that are not readily detected using HPLC-UV. An infrared image of the suspect bulk powder is shown in Figure 3a. A representative spectrum from the bulk gray region (Figure 3b) is consistent with that of the API reference standard (Figure 3c). A representative spectrum from the green region (Figure 3d) is consistent with that of an inorganic sulfate (Figure 3e); additional microchemical testing determined that the cation was sodium. The combined results indicated that the impurity was sodium sulfate, a compound used in the synthesis of this particular API that may not have been sufficiently rinsed out of the final product.

### 2.6. Analysis of Unknown Trace Powders

**Figure 4:**
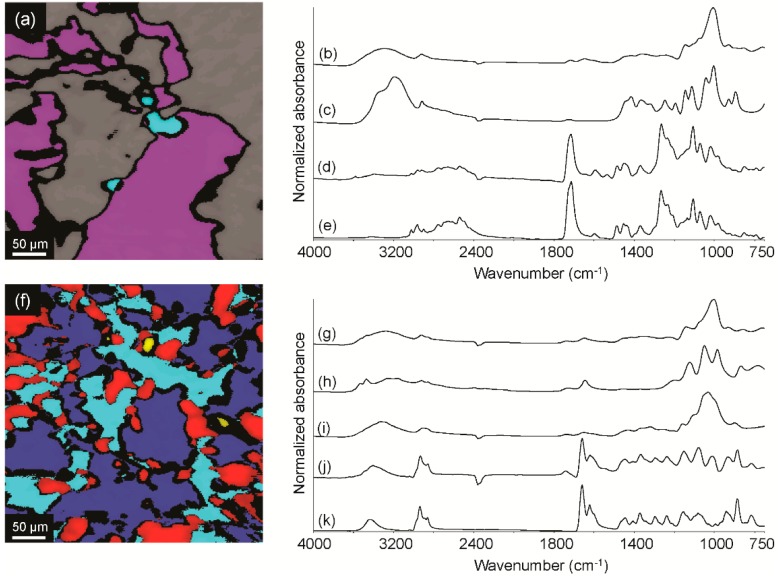
ATR-FT-IR image of Powder 1 (**a**); and representative spectra from the cyan (**b**); purple (**c**) and gray (**d**) regions compared to a pure compound reference spectrum of cocaine HCl (**e**); IR image of Powder 2 (**f**); and representative spectra from the cyan (**g**); red (**h**); blue (**i**) and yellow (**j**) regions compared to a pure compound reference spectrum of methandrostenolone (**k**).

Recently, two containers, each with a trace amount of powdery residue, were received as a single item. FCC was requested to examine the powders for controlled substances and steroids. Using GC/MS, the first powder was found to contain cocaine, a US Drug Enforcement Administration (DEA) Schedule II controlled substance. However, it could not be determined if the analyte was present as the salt or base form using this approach; charges/penalties for possession of cocaine HCl may be different than those of cocaine freebase, so differentiation of the two is often requested. Analysis of the powder using macro-ATR-FT-IR yielded a mixture spectrum, which indicated that the sample contained more than one ingredient. As a result, the powder was further examined using ATR-FT-IR spectroscopic imaging. The infrared image is shown in Figure 4a, and representative spectra from the cyan, purple and gray regions are shown in Figure 4b–d, respectively. Figure 4b was consistent with starch; Figure 4c was consistent with inositol, a common cutting agent; and Figure 4d was consistent with the cocaine HCl reference standard spectrum shown in Figure 4e, as indicated by the characteristic HCl absorption between 2600 and 2400 cm^−1^.

The macro-ATR-FT-IR spectrum of the second powder also exhibited evidence of a mixture, which warranted analysis using ATR-FT-IR spectroscopic imaging. The infrared image from the second powder is shown in Figure 4f, and representative spectra from the cyan, red and blue regions are shown in Figure 4g–i, respectively. Figure 4g was consistent with starch; Figure 4h was consistent with calcium hydrogen phosphate dihydrate; and Figure 4i was consistent with cellulose. Spectra from the yellow region exhibited absorptions associated with calcium hydrogen phosphate dihydrate and an unknown compound. Calcium hydrogen phosphate dihydrate was therefore spectrally subtracted from a representative spectrum from the yellow region, which yielded the spectrum shown in Figure 4j. Figure 4j was consistent with the methandrostenolone reference standard spectrum provided in Figure 4k; methandrostenolone is an anabolic steroid that is a DEA Schedule III controlled substance. Overall, the data indicate that the two residues were different, but they each contained a violative substance.

### 2.7. Analysis of a Tampered Antibiotic Ophthalmic Drug Solution

Over the last twenty years, the FCC has been requested to examine a large number of products suspected of being tampered with bleach (sodium hypochlorite), including beverages, infant formula, food and raw meat [30]. Sodium hypochlorite breaks down quickly in many matrices, which makes it difficult to detect the analyte directly. As a result, many analytical approaches aim toward detecting sodium hypochlorite indirectly from the presence of oxidizing agents, elevated pH, chloride and chlorate levels. Recently, FCC received antibiotic ophthalmic drops suspected of being adulterated with bleach along with a negative control; a portion of the control was later spiked with bleach for comparison.

**Figure 5:**
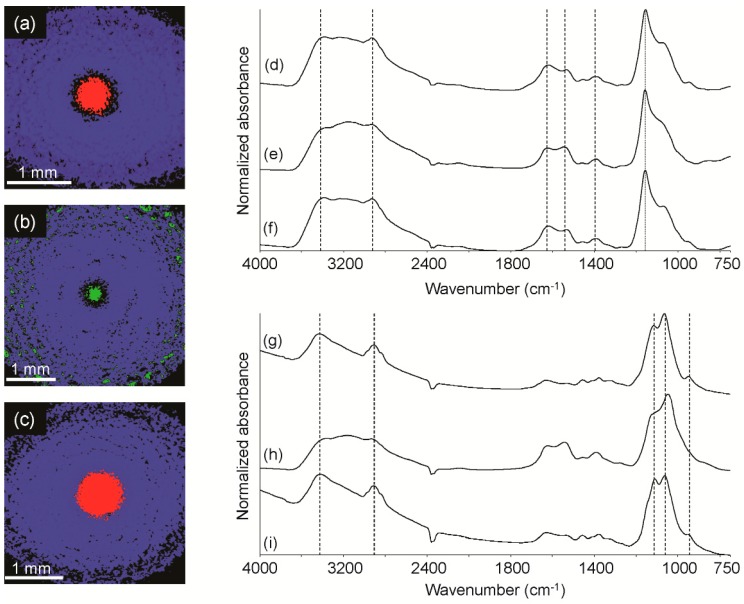
Reflection/absorption-IR images of the suspect dried residue (**a**); control dried residue (**b**) and control spiked with bleach dried residue (**c**); representative spectra from the blue region in the suspect image (**d**); control image (**e**) and control spiked with bleach image (**f**); representative spectra from the red region in the suspect image (**g**); green region in the control image (**h**) and red region in the control spiked with bleach image (**i**).

One drop each of the suspect, control and spiked control solution was deposited on a low-E microscope slide and left to air dry. Point mode micro-IR analysis of the dried residues yielded subtly different spectra, even within the same sample. Using this approach, it could not be determined whether these slight spectral differences were the result of more than one ingredient or simply point-to-point variation. As a result, infrared spectroscopic imaging was employed to collect a large number of spectra over each sample, which would either provide a representative sampling of a single ingredient or determine if more than one ingredient was present. The samples were examined using R/A mode because the diameter of each residue was larger than that of the IRE. Micro-infrared spectroscopic imaging analysis of the suspect (Figure 5a), control (Figure 5b) and spiked control (Figure 5c) residues indicated that a physical separation occurred as the solutions dried. Spectra extracted from the blue regions of the suspect (Figure 5d), control (Figure 5e) and spiked control (Figure 5f) images were consistent with each other; dashed lines are provided to demonstrate that no band shifting was observed. However, a representative spectrum extracted from the center of the suspect image (Figure 5g) was not consistent with that of the control (Figure 5h), but it was consistent with that of the spiked control (Figure 5i). Specifically, the suspect and spiked control spectra exhibited sloped baselines between 4000 and 2000 cm^−1^ and shifted bands compared to that of the control (indicated with dashed lines). Although bleach was not detected directly, these data indicate that (1) the suspect sample was not consistent with that of the control and (2) bleach could not be ruled out as an adulterant, which was consistent with elevated pH, chlorite and chlorate levels determined using ion chromatography, GC/MS and liquid chromatography-charged aerosol detection (LC-CAD). The suspect in this case was found guilty of first degree child assault and was sentenced to 40 years in prison.

## 3. Materials and Methods

Images were collected with a Spotlight 400 FT-IR Spectroscopic Imaging Microscope (PerkinElmer Inc., Waltham, MA, USA) in either R/A or ATR modes. Samples examined using R/A were deposited on a low-E infrared reflecting microscope slide. Samples examined using ATR were pressed into a pellet using a Carver Two Post manual hydraulic press (Carver Inc., Wabash, Indiana) and a Specac 13-mm die (Specac Ltd., Slough, UK). ATR analyses were conducted using a 1.5-mm radius germanium hemisphere IRE, which was lowered onto the sample prior to analysis. Collection parameters for the ATR measurements included 16 cm^−1^ resolution, 1 scan/pixel, 1.56 μm × 1.56 μm pixel resolution, 400 μm × 400 μm image area and 13.3-min collection time. Collection parameters for R/A measurements varied with each sample and are discussed in more detail throughout the text where appropriate.

Images in Figure 1, Figure 2, Figure 3, Figure 4 and Figure 5 were created using Spectrum Image-Spotlight software Version 400 R1.6.4.0394 (PerkinElmer). The infrared image was opened with the “compare correlation” option using a melamine cyanurate spectrum as a reference, which assigned a bright color to pixels with a high correlation to the selected spectrum and a dark color to pixels with a low correlation to the selected spectrum. This resulted in an image that displayed the distribution of melamine cyanurate in the image. While the same process was repeated for Figure 2, Figure 3, Figure 4 and Figure 5, these images required a few additional steps because they each contained more than one analyte. For example, the infrared image in Figure 2a was opened with the “compare correlation” option using a penicillin G sodium salt spectrum as a reference. In this case, a single-color (blue) correlation image was created instead of a bright/dark correlation image. Single-color correlation images were then created for penicillin G procaine salt hydrate (green) and penicillin G benzathine salt (red). The blue, green and red images were then overlaid to produce the final multi-component/multi-colored formulation image illustrated in Figure 2a. The same procedure was employed to create Figure 3, Figure 4 and Figure 5.

## 4. Conclusions

Based on the current literature and results of this study, infrared spectroscopic imaging has proven to be useful for examining a wide range of multi-component forensic casework samples. Regarding this study, the technique was effective for examining animal tissue sections for toxic inclusions, multi-component drugs/dietary supplements, an antibiotic with an API present as several different salt forms, an adulterated bulk API, unknown trace powders for illicit drugs and an ophthalmic solution suspected of being adulterated with bleach. The results described herein were not intended to demonstrate that this technique can be used in a vacuum, but rather were intended to demonstrate that it can be useful in conjunction with other methods early in an investigation and as a primary method or as a confirmatory method to provide a more accurate overall assessment of a suspect product.

## Figures and Tables

**Table 1:** Combination drugs and multi- active pharmaceutical ingredient (API) dietary supplements examined by the Forensic Chemistry Center (FCC) using FT-IR spectroscopic imaging.

Combination Drugs	Dosage (s)	Active Ingredient (s)
Anti-malarial	250/100 mg	atovaquone, praguanil HCl
Cholesterol	80/10 mg, 40/10 mg	ezetimbe, simvastatin
HIV	300/150 mg	lamivudine, zidovudine
HIV	300/200 mg	tenofovir disoproxil fumarate, emtricitibine
Hypertension	320/25, 320/12.5 mg	valsartan, hydrochlorothiazide
Hypertension	320/10, 160/10 mg, 160/5 mg	valsartan, amlodipine besylate
Hypertension	100/12.5 mg, 50/12.5 mg	losartan potassium, hydrochlorothiazide
Hypertension	160/150 mg	valsartan, aliskiren
Hypertension	40/12.5 mg	olmesartan medoxomil, hydrochlorothiazide
Hypertension	300/12.5 mg, 150/25 mg, 150/12.5 mg	aliskiren hemifumarate, hydrochlorothiazide
Hypertension/Angina	40/5 mg	atorvastatin, amlodipine besylate
Pain Reliever	300/50/40 mg	acetaminophen, butalbital, caffeine
Pain Reliever	325/10 mg, 325/5 mg	acetaminophen, oxycodone HCl
Pain Reliever	325/10 mg	acetaminophen, hydrocodone bitartrate
Parkinson’s	100/100/200 mg	carbidopa, levodopa, entacapone
Type 2 Diabetes	1000/50 mg, 500/50 mg	metformin HCl, sitagliptin
**Dietary Supplements**	**Ingredient (s)**	
1	aegeline, higenamine HCl, caffeine	
2	aegeline, caffeine	
3	ascorbic acid, n-acetyl-l-cysteine, p-aminobenzoic acid	
4	ubiquinone, starch, inorganic stearate, microcrystalline cellulose, inorganic carbonate	
5	creatine, starch, inorganic stearate	
6	1,3 dimethylpentylamine, glaucine, cellulose, talc	
7	caffeine, cellulose, inorganic carbonate, inorganic phosphate	
8	phenolphthalein, tetracaine HCl, fenfluramine, propanolol HCl, sucrose, dextrin, inorganic oxalate	
9	phenolphthalein, acetaminophen, propanolol HCl	
10	tadalafil, gelatin	

## Abbreviations

The following abbreviations are used in this manuscript:

FCC: Forensic Chemistry Center
API: Active pharmaceutical ingredient
FT-IR: Fourier transform infrared
ATR: Attenuated total reflection
R/A: Reflection/absorption
IRE: Internal reflection element
GC/MS: Gas chromatography with mass spectrometric detection
LC/MS: Liquid chromatography with mass spectrometric detection
LC/UV: Liquid chromatography with ultraviolet detection
LC-CAD: Liquid chromatography-charged aerosol detection
USP: United States Pharmacopoeia
